# The p97 segregase cofactor Ubxn7 facilitates replisome disassembly during S-phase

**DOI:** 10.1016/j.jbc.2022.102234

**Published:** 2022-07-04

**Authors:** Zeynep Tarcan, Divyasree Poovathumkadavil, Aggeliki Skagia, Agnieszka Gambus

**Affiliations:** Institute of Cancer and Genomic Sciences, Birmingham Centre for Genome Biology, College of Medical and Dental Sciences, University of Birmingham, Vincent Drive, Birmingham United Kingdom

**Keywords:** termination of DNA replication, DNA replication, p97 segregase, ubiquitin, *Xenopus laevis*, CMG, Cdc45, Mcm2-7 hexamer, and GINS, DMSO, dimethyl sulfoxide, FDR, false discovery rate, IP, immunoprecipitate, MS, mass spectrometry, UIM, ubiquitin-interacting motif

## Abstract

Complex cellular processes are driven by the regulated assembly and disassembly of large multiprotein complexes. While we are beginning to understand the molecular mechanism for assembly of the eukaryotic DNA replication machinery (replisome), we still know relatively little about the regulation of its disassembly at replication termination. Recently, the first elements of this process have emerged, revealing that the replicative helicase, at the heart of the replisome, is polyubiquitylated prior to unloading and that this unloading requires p97 segregase activity. Two different E3 ubiquitin ligases have now been shown to ubiquitylate the helicase under different conditions: Cul2^Lrr1^ and TRAIP. Here, using *Xenopus laevis* egg extract cell-free system and biochemical approaches, we have found two p97 cofactors, Ubxn7 and Faf1, which can interact with p97 during replisome disassembly during S-phase. We show only Ubxn7, however, facilitates efficient replisome disassembly. Ubxn7 delivers this role through its interaction *via* independent domains with both Cul2^Lrr1^ and p97 to allow coupling between Mcm7 ubiquitylation and its removal from chromatin. Our data therefore characterize Ubxn7 as the first substrate-specific p97 cofactor regulating replisome disassembly in vertebrates and a rationale for the efficacy of the Cul2^Lrr1^ replisome unloading pathway in unperturbed S-phase.

DNA replication is one of the most fundamental processes in life and its faultless execution is essential for normal cell fate. Until recently, the final stage of eukaryotic DNA replication, the termination stage, was mostly unexplored. DNA replication initiates from thousands of replication origins, the positions within the genome where replicative helicases become activated and start unwinding DNA. These then move in opposite directions away from each other, creating two DNA replication forks. The replicative helicase is composed of Cdc45, Mcm2-7 hexamer, and GINS complex (CMG complex) (1) and is positioned at the tip of replication forks forming a platform for replisome assembly (2). Once established, the replication forks replicate chromatin until they encounter forks coming in opposite directions from neighboring origins. At this point the termination of replication forks takes place, with removal of the replisome from fully duplicated DNA being its final stage (3). In higher eukaryotes (*Xenopus laevis* egg extract, *Caenorhabditis elegans* embryos, mouse embryonic fibroblasts, and human cells), replisome removal in S-phase is driven by the Cul2^Lrr1^ ubiquitin ligase, which ubiquitylates Mcm7 within the terminated CMG helicase complex with lysine 48 (K48)-linked ubiquitin chains (4). The modified CMG is then recognized by the p97 segregase and removed from chromatin allowing for disassembly of the whole replisome built around the helicase (5). Any helicase complexes that fail to be unloaded in S-phase are alternatively unloaded in mitosis. Disassembly of these complexes in mitosis also depends on p97 segregase function, but this time requires the E3 ubiquitin ligase TRAIP (6). Consequently, disassembly in mitosis is driven by alternative species of ubiquitin chains that decorate Mcm7, namely K6- and K63-linked ubiquitin chains. TRAIP ubiquitin ligase can act also during S-phase: it interacts with the replisome and either ubiquitylates CMGs that converge at interstrand crosslinks or ubiquitylates a protein crosslinked to DNA (DPC) that blocks progression of the replication fork (7, 8).

p97 segregase (also referred to as VCP, Cdc48, CDC-48, and Ter94) is a hexameric AAA+ ATPase family member that uses the free energy of ATP binding and hydrolysis to extract ubiquitylated protein targets from stable protein complexes, chromatin, or membranes. As a result, p97 is essential for protein homeostasis in the cell and the dynamic behavior of a multitude of multiprotein assemblies (9). The substrate specificity of p97 recognition is believed to be achieved by a number of regulatory cofactors (reviewed in (10)). In *C. elegans* embryos, the CDC-48 cofactors UFD-1/NPL-4 and UBXN-3 (Faf1 in higher eukaryotes) were shown to be required for replisome removal from chromatin in both S-phase and in mitosis (4, 11). UFD-1/NPL-4 form a heterodimer, essential for most chromatin-related roles of p97, while UBXN-3 provides higher substrate specificity. Interestingly, Ufd1/Npl4 were also shown to interact with p97 and the replisome during replication termination in *Xenopus* egg extract (4). However, while Ufd1/Npl4 are evolutionarily conserved and well characterized, the number and variability of the minor, substrate specific, cofactors of p97 grows through evolution, reflecting the increasing complexity of p97 regulation. So far, roughly three times more cofactors have been identified in mammals than in *C. elegans* (4, 12). Importantly, additional cofactors of p97, which provide substrate specificity towards the terminated replisomes, are as yet to be determined in vertebrates.

Here, we sought to identify p97 cofactors that are facilitating replisome disassembly during S-phase. While we were able to identify two new cofactors for this process, Ubxn7 and Faf1, our findings revealed that the Ubxn7 cofactor specifically is crucial for efficient and fast disassembly of replisomes, as it creates bridges between the essential factors of this process: Cul2^Lrr1^, ubiquitylated Mcm7, and the p97 complexes.

## Results

### Identification of p97 cofactors acting during unloading of replicative helicase

**Figure 1:**
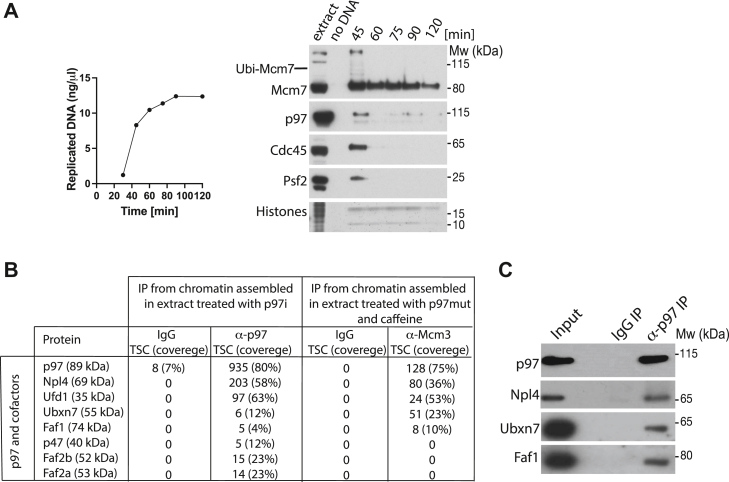
**Cofactors interacting with p97 on replicating chromatin in *Xenopus laevis* egg extract.***A*, chromatin binding of p97 follows replication fork components. A replication reaction was set up in *X. laevis* egg extract and synthesis of nascent DNA was followed by incorporation of radioactive α-P^32^dATP into newly synthesized DNA (*left*). At the same time, chromatin was isolated during the replication reaction at indicated time points after sperm DNA addition. Sample without DNA addition was processed in parallel to provide a chromatin specificity control. Histones at the *bottom* of the PAGE gel were stained with colloidal Coomassie for loading and sample purity control. Chromatin samples were analyzed by Western blotting with indicated antibodies (*right*). *B*, Ubxn7 and Faf1 are identified as cofactors interacting with both p97 and terminated replisome. Interphase egg extract was supplemented with p97i (NMS873) and chromatin was isolated in late S-phase when high levels of terminated replisomes are accumulated on chromatin. Protein complexes were released from chromatin by Benzonase treatment and proteins interacting with p97 segregase were analyzed by mass spectrometry. Identified putative interactors were screened to find known and potential p97 cofactors. Obtained data were compared with the mass spectrometry results of interactors of terminated replisomes published previously (4). Total spectral count is presented with protein coverage in the brackets. Only p97 and its cofactors are presented. Other proteins identified in p97 IP are included in Table S1. *C*, verification of p97 interaction with Ubxn7 and Faf1 on chromatin. A small proportion of input and immunoprecipitated sample from p97 IP described in (*B*) was analyzed by Western blotting with indicated antibodies.

Using the *X. laevis* egg extract model system, we have previously shown that the unloading of terminated replicative helicases during S-phase depends on formation of K48-linked ubiquitin chains on the Mcm7 subunit of the CMG helicase by Cul2^Lrr1^ ubiquitin ligase (5). Such modified Mcm7 is in turn recognized and unfolded by p97. We therefore first confirmed that p97 interacts with replicating chromatin in *X. laevis* egg extract with kinetics similar to replication fork components (Fig. 1*A*). We found that p97 is a highly abundant protein in *Xenopus* egg extract (13), and only a small proportion of it interacts with chromatin during DNA replication, with the interaction peaking during the exponential stage of replication, when large numbers of replication forks are moving through the chromatin and converging during termination (Fig. 1*A*). To determine the portfolio of p97 cofactors that interact with p97 during DNA replication termination in egg extract and which may direct p97 to the terminated replisome, we aimed to immunoprecipitate (IP) p97 from a chromatin fraction and analyze its interactors. Firstly, we blocked replisome disassembly by inhibiting p97 activity with NMS-873 (p97i), which is a highly specific allosteric inhibitor of p97 ATPase activity binding to p97 D2 domain (14). We have shown previously that inhibition of p97 ATPase activity stops replisome unloading from chromatin (4). Critically, this treatment does not stop p97 from interacting with substrates or chromatin (Fig. S1*A*) and should stabilize p97–substrate complexes on chromatin as the substrates cannot be processed. Subsequently, we isolated chromatin with accumulated terminated replisomes, immunoprecipitated p97 and analyzed interacting factors by mass spectrometry (MS). Such analysis revealed numerous components of replication machinery interacting with p97, including those which reside in the replisome built around the CMG helicase but also other DNA replication and DNA damage repair factors (Table S1). In order to focus our analysis on potential p97 cofactors, which direct p97 specifically to the terminated replisomes, results from the p97 interactome were compared with those of an Mcm3 IP, which was performed in conditions blocking replisome disassembly. Briefly, the extract was replicated in the presence of a dominant negative ATPase-dead mutant of p97, described previously, and Mcm3 was immunoprecipitated to isolate terminating replisomes (4, 13). This comparison allowed us to determine which of the p97 cofactors identified in the p97 immunoprecipitation are also interacting with the terminated replisomes, as we appreciate that p97 does have other substrates on replicating chromatin (Fig. 1*B*). In doing this, we identified both major cofactors Ufd1 and Npl4, which are known to facilitate chromatin functions of p97 segregase and were shown previously by us and others to act in replisome disassembly during S-phase (4, 15). Interestingly, only two minor cofactors were identified to interact with both the replisome and p97: Faf1 and Ubxn7 (human UBXD7). Both of these factors have been shown previously to interact preferentially with p97 when in complex with Ufd1/Npl4 (16). To support this finding, we first confirmed by p97 IP and Western blotting that p97 can indeed interact with Ubxn7 and Faf1 on S-phase chromatin when replisome disassembly is blocked (Fig. 1*C*).

The finding of Faf1 was somewhat unsurprising as it is already known to play a role in maintaining replication fork stability in *C. elegans* and human cell lines (17), and indeed, the *C. elegans* homolog of Faf1 (UBXN-3) is essential for replisome unloading in S-phase and in mitotic prophase (4, 11). In contrast, this is the first time that Ubxn7 has been implicated in the process of replisome unloading. What is already known about Ubxn7, is that inhibition of the human homolog UBXD7 in human cells leads to hyperaccumulation of DNA damage sensors after UV damage (18), although it is best known as a regulator of degradation of the hypoxia inducible factor Hif1α (19). Like Faf1, Ubxn7 belongs to the ubiquitin-associated (UBA)–ubiquitin regulatory X (UBX) family of p97 cofactors. This means that it interacts with ubiquitylated proteins *via* its UBA domain and with p97 *via* its UBX domain. It also contains a UAS domain of unknown function and an ubiquitin-interacting motif (UIM) (Fig. S2*A*) (19). Interestingly, while targeting Hif1α for degradation, UBXD7 simultaneously interacts with active (neddylated) cullin ligase Cul2^VHL^ through its UIM domain and with p97 through its UBX domain (Fig. S2, *A* and *B*) (20). Given that the same factors appear to be involved in replisome disassembly, we hypothesized that the association of p97 with its cofactor Ubxn7 could provide a mechanism by which p97 targets Mcm7, ubiquitylated with K48-linked ubiquitin chains, synthesized by Cul2^Lrr1^.

### Ubxn7 stimulates efficient replisome disassembly

**Figure 2:**
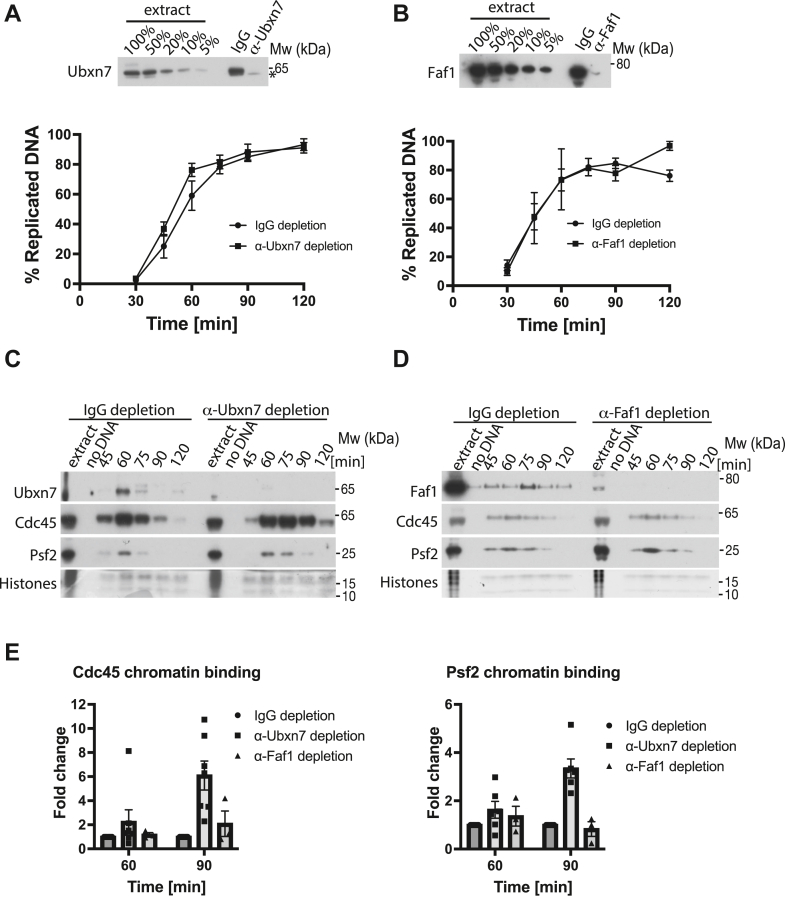
**Ubxn7 facilitates terminated replisome unloading.***A*, Ubxn7 is not required for DNA replication. The remaining level of Ubxn7 upon Ubxn7 immunodepletion was analyzed by Western blotting. The *asterisk* indicates a nonspecific band (*top*). The ability of Ubxn7-immunodepleted extract to synthesize nascent DNA was analyzed by incorporation of radioactive α-P^32^dATP into newly synthesized DNA (n = 6) (*bottom*). *B*, Faf1 is not required for DNA replication. The remaining level of Faf1 upon Faf1 immunodepletion was analyzed by Western blotting (*top*). The ability of Faf1-immunodepleted extract to synthesize nascent DNA was analyzed by incorporation of radioactive α-P^32^dATP into newly synthesized DNA (n = 3) (*bottom*). *C*, Ubxn7 depletion delays terminated replisomes disassembly. Chromatin was isolated during the replication reaction time course in IgG-depleted and Ubxn7-depleted extract. Chromatin samples were analyzed as in Figure 1*A* with indicated antibodies. Representative experiment out of n = 7. Another repeat of this experiment is presented in Fig. S3*C*. *D*, Faf1 depletion does not impact terminated replisomes disassembly. Chromatin was isolated during the replication reaction time course in IgG-depleted and Faf1-depleted extract. Chromatin samples were analyzed as in Figure 1*A* with indicated antibodies. Representative experiment out of n = 3. *E*, the fold increase of Cdc45 and Psf2 signal on chromatin in Ubxn7- or Faf1-depleted extracts at indicated time points was quantified in comparison to IgG depletion. For Ubxn7-depleted extract n = 7 for Faf1-depleted extract n = 3. The mean value is presented with all individual points and with SEM as error bars.

To investigate the importance of Faf1 and Ubxn7 for replisome disassembly during S-phase, we decided to immunodeplete each protein independently from the egg extract and determine the consequences for replisome unloading. To do this, we have raised antibodies against *X. laevis* Ubxn7 and Faf1 (Fig. S7). Both Faf1 and Ubxn7 could be efficiently immunodepleted from egg extracts to less than 5% of total protein (Figs. 2, *A* and *B* and S3*B*). Neither depletion inhibited the synthesis of nascent DNA in a number of independent immunodepletions (Fig. 2, *A* and *B*), suggesting that neither are essential for DNA synthesis completion in the egg extract. Importantly, neither Ubxn7 nor Faf1 immunodepletion affected the level of each other (Fig. S3*A*). We then followed proteins on chromatin during a replication reaction in IgG-depleted and Ubxn7- or Faf1-depleted extracts. Interestingly, while immunodepletion of Faf1 had a very minor effect on replisome unloading during S-phase (Fig. 2, *D* and *E*), immunodepletion of Ubxn7 reproducibly delayed unloading of replisomes (Cdc45, Psf2) in independently immunodepleted extracts (Figs. 2, *C* and *E* and S3*C*), suggesting that although Ubxn7 is not essential for replisome disassembly, it does regulate the efficiency of this process.

### Importance of Ubxn7 for replisome disassembly

**Figure 3:**
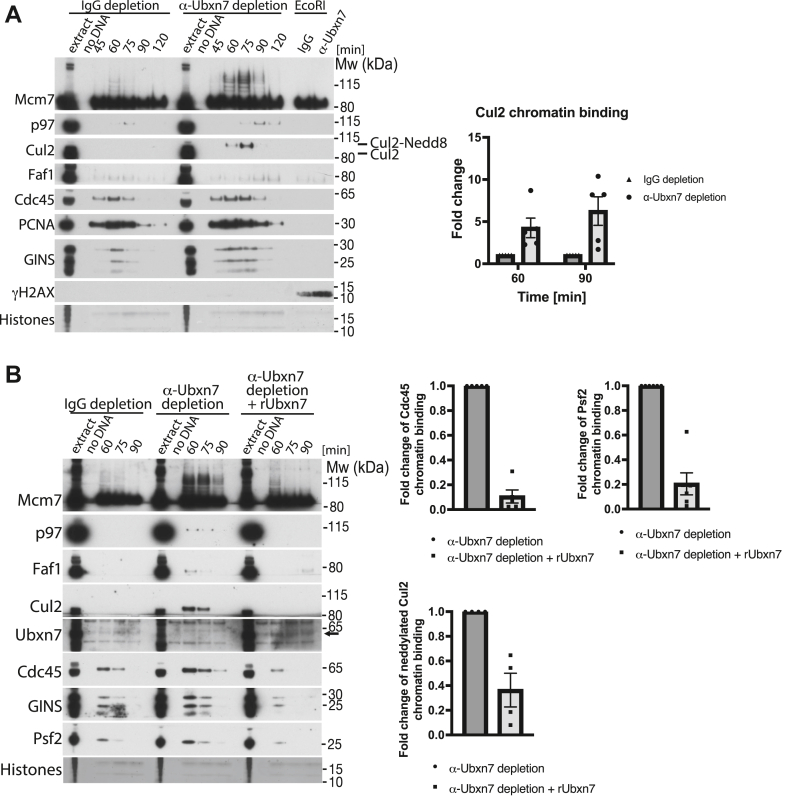
**Ubxn7 depletion leads to accumulation of ubiquitylated Mcm7 and active Cul2**^**Lrr1**^**on chromatin.***A*, chromatin was isolated during the replication reaction time course in IgG-depleted and Ubxn7-depleted extract. Chromatin samples were analyzed as in Figure 1*A* with indicated antibodies. Representative experiment is presented (*left*). The fold increase of Cul2 signal on chromatin in Ubxn7-depleted extract at indicated time points was quantified in comparison to IgG depletion (n = 5) (*right*). The mean value is presented with all individual points and with SEM as error bars. *B*, chromatin was isolated during the replication reaction time course in IgG-depleted, Ubxn7-depleted, and Ubxn7-depleted extract supplemented with recombinant Ubxn7 at 10 μg/ml. Samples analyzed as in (*A*). Representative experiment (*left*). The rescue of the phenotypes by rUbxn7 was quantified in four experiments and the fold downregulation of Cdc45, Psf2 Cdc45 and Cul2 retainment presented as a mean value with SEM (*right*).

Next, we wanted to understand how the depletion of Ubxn7 delays the unloading of the replisomes in the egg extract. Induction of replication stress, which affects progression of replication forks, can lead to transient accumulation of replisomes on chromatin. However, Ubxn7 depletion did not lead to the accumulation of DNA damage-associated replication stress, as determined by γH2AX immunoblotting (Fig. 3*A*), nor the phosphorylation of Chk1 (Fig. S3*D*). Taken together, this demonstrates that accumulation of replisomes on chromatin in Ubxn7-depleted extract is not due to the effects of replication stress.

We next analyzed how Ubxn7 depletion affects the levels of Mcm7 ubiquitylation and chromatin-bound Cul2 and p97 (Fig. 3*A*). Interestingly, while the levels of p97 and Faf1 on chromatin were slightly increased by Ubxn7 depletion (Fig. 3*A*), Cul2 markedly accumulated on chromatin in its active neddylated form (Fig. 3*A*, neddylated Cul2 runs at a higher molecular size on the gel—compare size of unneddylated Cul2 in egg extract and neddylated on chromatin). Similarly, ubiquitylated Mcm7 also accumulated on chromatin, modified with long ubiquitin chains.

We then decided to ensure that these phenotypes are actually caused by immunodepletion of Ubxn7 rather than an unidentified protein recognized by Ubxn7 antibody, through supplementing immunodepleted extract with recombinant Ubxn7. As shown in Figure 3*B*, addition of recombinant Ubxn7 could rescue the delayed unloading of CMG components (Cdc45 and Psf2), the accumulation of Cul2 on chromatin, and the accumulation of long-chain modified Mcm7 (Fig. 3*B*). These results suggest that Ubxn7 may be fine-tuning the process of replisome unloading, acting as a bridge between Cul2^Lrr1^, p97, and their shared substrate Mcm7. This bridging allows for fast and efficient processing of Mcm7 ubiquitylated with relatively short ubiquitin chains. Without Ubxn7, Cul2^Lrr1^ recognizes terminated CMG and ubiquitylates Mcm7, but this ubiquitylated Mcm7 is not recognized and processed quickly by p97 due to lack of Ubxn7. Cul2^Lrr1^ stays therefore associated with Mcm7 for a longer time (we can observe this as accumulation of active Cul2 on chromatin), resulting in synthesis of longer ubiquitin chains on Mcm7. Although this most likely enables eventual recruitment of p97, the process is less efficient.

### Regulation of Ubxn7 during DNA replication in egg extract

**Figure 4:**
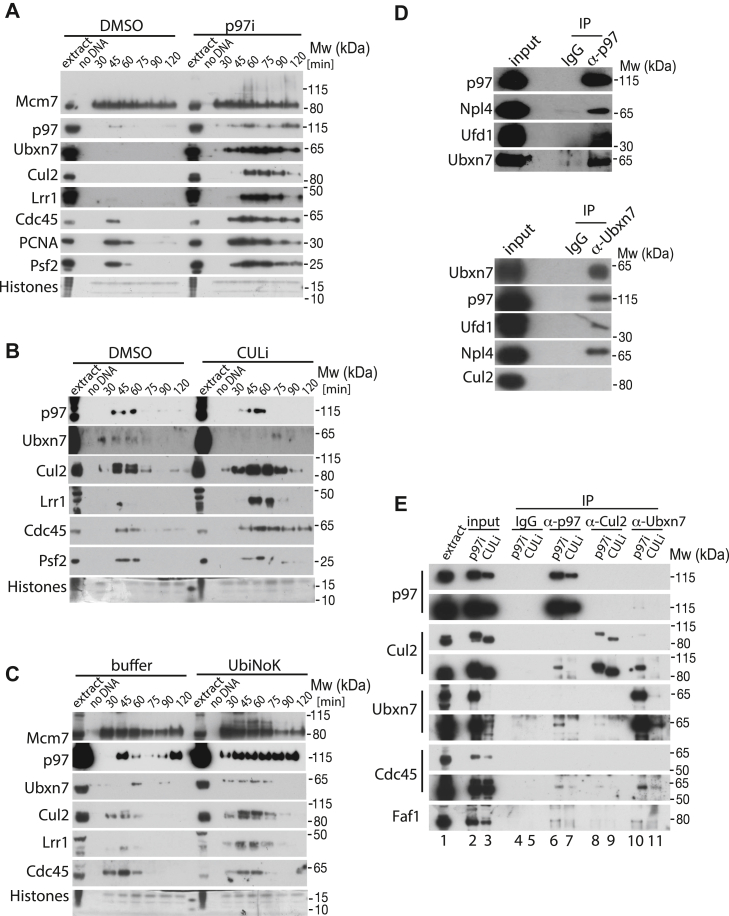
**Regulation of Ubxn7 chromatin binding.***A*, p97 and Ubxn7 accumulate on chromatin upon p97 activity inhibition. Interphase egg extract was supplemented with DMSO or p97i and chromatin was isolated during the replication reaction. Chromatin samples were analyzed as in Figure 1*A*. Representative experiment is presented here and quantification over n = 3 in Fig. S4*A*. *B*, p97 and Ubxn7 decrease on chromatin upon cullin activity inhibition. Interphase egg extract was supplemented with DMSO or CULi and chromatin was isolated during the replication reaction. Chromatin samples were analyzed as in (*A*). Representative experiment is presented here and quantification of n = 4 in Fig. S4*B*. *C*, p97 and Ubxn7 accumulate on chromatin upon inhibition of polyubiquitylation. Interphase egg extract was supplemented with LFB1/50 buffer or 6HIS-UbiNoK and chromatin was isolated during the replication reaction. Chromatin samples were analyzed as in (*A*). Representative experiment is presented here and quantification of n = 3 in Fig. S4*C*. *D*, Ubxn7 interacts with p97 but not with Cul2 in egg extract. Ubxn7 or p97 were immunoprecipitated from egg extract. Interacting partners were analyzed by Western blotting with indicated antibodies. *E*, interaction between Ubxn7, p97, and Cul2 is disrupted when neddylation of Cul2 is inhibited. Interphase egg extract was supplemented with CULi (MLN4924) or p97i (NMS873). Chromatin was isolated in late S-phase when a high level of post-termination replisomes accumulated on chromatin, protein complexes were released from chromatin by Benzonase treatment and p97, Cul2, or Ubxn7 immunoprecipitated from the chromatin proteome. Immunoprecipitated samples were analyzed by Western blotting with indicated antibodies. Short and long exposures for each of the immunoprecipitated proteins are presented. DMSO, dimethyl sulfoxide.

To determine how Ubxn7 is regulated during replication termination and replisome disassembly in the egg extract, we analyzed its pattern of chromatin binding during a replication reaction in egg extract. Throughout normal, unchallenged replication, Ubxn7 transiently interacts with chromatin with timing concomitant to that of replication fork presence and Cul2^Lrr1^ (Figs. 4, *B* and *C* and S4, *A*–*C* (dimethyl sulfoxide [DMSO] control)). Inhibition of replisome unloading with p97 ATPase inhibitor led to an accumulation of not only CMG and Cul2^Lrr1^ on chromatin as expected (4) but also of Ubxn7 and p97 (Figs. 4*A* and S4*A*). We then analyzed the Ubxn7 chromatin-binding pattern upon inhibition of cullin ligases activity. CULi MLN4924 acts through inhibition of Nedd8 activating enzyme NAE and therefore inhibiting neddylation of substrates (21). As members of the cullin family of ubiquitin ligases are the main substrate of neddylation in cells, MLN4924 is primarily inhibiting all cullin activity. We have shown previously that, in *X. laevis* egg extract, inhibition of cullin activity during DNA replication inhibits Mcm7 ubiquitylation and replisome unloading from chromatin (5). While inhibition of cullin activity led to accumulation on chromatin of CMG and Cul2^Lrr1^, as we reported previously (4), levels of chromatin-bound p97 and Ubxn7 were reduced (Figs. 4*B* and S4*B*). This result suggests that the key determinants of p97 chromatin binding during DNA replication in the egg extract are either cullin-driven ubiquitylation of substrates and/or neddylation of cullins.

Finally, we decided to inhibit the polyubiquitylation of all the potential substrates of p97 during DNA replication by supplementing the extract with a chain terminating mutant of ubiquitin: 6xHIS-UbiNoK. Interestingly, despite inhibition of polyubiquitylation by 6xHIS-UbiNoK, as observed through accumulation of di-monoubiquitylated forms of Mcm7 on chromatin, we can reproducibly observe a higher level of p97 and Ubxn7 binding to chromatin (Figs. 4*C* and S4*C*). Importantly, neither inhibition of cullins, nor global polyubiquitylation, affects the extracts’ ability to replicate DNA (Fig. S4, *B* and *C*). Our findings therefore suggest that Ubxn7 behaves like a p97 cofactor and follows the same patterns of chromatin interaction. Moreover, p97 segregase is not directed to chromatin and terminated replisomes just simply through interaction with polyubiquitylated substrates, as inhibition of polyubiquitylation does not prevent p97 and Ubxn7 from binding to chromatin (Figs. 4*C* and S4*C*). It is likely therefore that treatment of replicating extract with the cullin neddylation inhibitor (MLN4924) (Figs. 4*B* and S4*B*) downregulates chromatin binding of p97 and Ubxn7 because neddylated cullins provide a binding platform for Ubxn7.

To support this hypothesis further, we confirmed that Ubxn7 can interact with p97 not only on chromatin (Fig. 1*C*) but also in the egg extract (cytoplasm) (Fig. 4*D*). However, we could not detect an interaction between Ubxn7 and Cul2 in the egg extract (Fig. 4*D*), as Cul2 is present in the cytoplasmic extract in its inactive, unneddylated form. We went on to perform reciprocal immunoprecipitations of p97, Cul2, and Ubxn7 from chromatin where post-termination replisomes were accumulated due to inhibition of cullin neddylation (CULi) or p97 activity (p97i) (Fig. 4*E*). The level of inhibition achieved in the samples can be judged by the status of Cul2 in the input; while Cul2 is present in the extract mainly in its inactive, unneddylated form (Fig. 4*E*, lane 1), it accumulates on chromatin during termination upon inhibition of p97 activity mostly in its active, neddylated form (Fig. 4*E*, lane 2). CULi treatment, however, leads to Cul2 accumulation on chromatin in its inactive unneddylated form (Fig. 4*E*, lane 3). The dramatic absence of Ubxn7 on chromatin upon neddylation inhibition, despite the presence of p97 and Faf1 in this input (Fig. 4*E*, lane 3), clearly suggests that Ubxn7 binding to chromatin strongly depends on cullin neddylation. When present in the chromatin input, Ubxn7 could coimmunoprecipitate neddylated Cul2, Faf1, and a little of p97 (Fig. 4*E*, lane 10). p97 could interact with Ubxn7, Faf1, and neddylated Cul2, but this can only be detected when replisomes have accumulated in their ubiquitylated form due to inhibition of p97 activity (Fig. 4*E*, compare lane 6–7). Similarly, despite immunoprecipitation of equal quantities of neddylated and unneddylated Cul2 from each sample, Cul2 could only interact with Ubxn7 when in its neddylated form on p97i treated chromatin (Fig. 4*E*, lane 8 and 9). Reassuringly, all three, p97, Cul2 and Ubxn7, could coimmunoprecipitate a little of the component of the terminated replisome Cdc45. Altogether, these experiments suggest that Ubxn7, although being a p97 cofactor, is recruited to chromatin during the termination reaction through its interaction with neddylated Cul2. Moreover, Faf1 is likely to form a common complex with p97 and Ubxn7 as we can see it interacting with Ubxn7 when present on chromatin (Fig. 4*E*, lane 10).

### Ubxn7 bridges Cul2^Lrr1^ and p97 through its UIM and UBX domains

Our results suggest that, analogously to Hif1α regulation, Ubxn7 acts as a bridge between Cul2^Lrr1^, its substrate Mcm7, and the p97 segregase complex. To explore this idea in more detail, we decided to make use of separation-of-function mutants of Ubxn7 that cannot interact with p97 (UBX domain mutated, rUbxn7^ΔUBX^) or Cul2 (UIM domain mutated, rUbxn7^ΔUIM^) (Fig. S2, *A* and *C*). In human cells, the P459G mutation abolishes interaction with p97, while S297A abolishes interaction with neddylated Cul2, while not affecting p97 interaction (20). Moreover, L290, A293 and S297 were found to be the most conserved amino acids in UIMs of several human proteins (22). We have therefore mutated corresponding P458G in the *X. laevis* Ubxn7 sequence to create rUbxn7^ΔUBX^ and the corresponding L286E/A289Q/S293A residues to create rUbxn7^ΔUIM^ (Fig.S5*A*). While we were able to confirm that rUbxn7^ΔUBX^ cannot interact with p97 in the egg extract (Fig. S5*B*), Ubxn7 and Cul2 do not interact in the egg extract (cytoplasm) and so it is not easy to verify whether the rUbxn7^ΔUIM^ mutation disrupts this. We did observe, however, that adding a high concentration of recombinant Ubxn7^ΔUIM^ mutant to normal egg extract with endogenous Ubxn7 present (mimicking overexpression experiments), caused a substantial increase in active, neddylated Cul2 on chromatin. This was not observed upon addition of wt rUbxn7 or rUbxn7^ΔUBX^ mutants (Fig. S5*C*). This suggests that outcompeting endogenous Ubxn7 with a mutant that cannot interact with neddylated Cul2 reproduces the phenotype of Ubxn7 immunodepletion, that is, increased and prolonged association of Cul2 with chromatin (Fig. 3*A*). Importantly, addition of neither protein affected the synthesis of nascent DNA (Fig. S5*D*). Altogether, these results suggest that Ubxn7^ΔUIM^ is defective in binding to neddylated Cul2.

**Figure 5:**
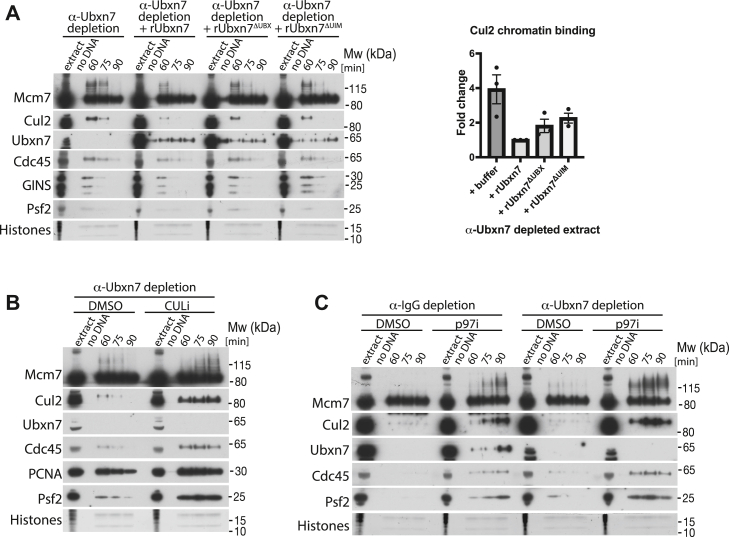
**Ubxn7 bridges Cul2**^**Lrr1**^**and p97 complexes leading to efficient unloading of ubiquitylated Mcm7.***A*, both UIM and UBX domains of Ubxn7 are important for its functions. Ubxn7 depleted extract was supplemented with recombinant Ubxn7 or point mutants that disrupt UBX or UIM domain functions (Ubxn7^ΔUBX^ and Ubxn7^ΔUIM^, respectively). Chromatin was isolated at indicated time points and analyzed as in Figure 1*A*. Representative experiment is presented (*left*). The level of Cul2 chromatin binding at 60 min time point was quantified over three experiments and the fold rescue of the Cul2 accumulation in comparison to Ubxn7 depleted extract is presented (*right*). Individual values, mean, and SEM are shown. CMG unloading is quantified in Fig. S6*B*. *B*, cullin activity is needed for replisome unloading in absence of Ubxn7. Ubxn7-depleted extract was supplemented with DMSO or CULi and chromatin samples isolated during the replication reaction and analyzed as in (*A*). *C*, the chains built on Mcm7 in Ubxn7 depleted extract are shorter than those built upon p97 activity inhibition. IgG- or Ubxn7-depleted extracts were optionally supplemented with p97i. Chromatin samples were analyzed at indicated time points as in (*A*). CMG, Cdc45, Mcm2-7 hexamer, and GINS; DMSO, dimethyl sulfoxide; UIM, ubiquitin-interacting motif.

To further test functionality of these mutants, Ubxn7 immunodepleted extract was supplemented with either WT or mutant Ubxn7. Neither addition to immunodepleted extract affected the synthesis of nascent DNA (Fig. S6*A*). While addition of WT Ubxn7 could support timely unloading of CMG from chromatin and prevent excessive accumulation of Cul2 on chromatin and long ubiquitin chain formation on Mcm7, neither of the two mutants could fully rescue the Ubxn7 immunodepletion phenotypes (Figs. 5*A* and S6*B*). This indicates that both domains are important for Ubxn7 function during replisome disassembly. Altogether, these data suggest that through its UBX and UIM domains, Ubxn7 can indeed bridge Cul2^Lrr1^ and the p97 complex and that binding to neddylated Cul2^Lrr1^ through its UIM domain is especially important for restricting Cul2 activity and/or stimulating its dissociation from the terminated replisome during replication termination.

### Unrestricted Cul2 activity allows for Mcm7 unloading in Ubxn7 depleted extract

Our aforementioned results show that upon Ubxn7 depletion, we observe accumulation on chromatin of active, neddylated Cul2^Lrr1^ and ubiquitylated forms of Mcm7. It is likely therefore that continuous growth of the length of chains on Mcm7 finally leads to p97 recognition and unloading. To test that it is Cul2^Lrr1^ and not a different ubiquitin ligase (*e.g.*, TRAIP) synthesizing these long chains, we blocked Cul2 activity using CULi in IgG- and Ubxn7-depleted extracts (Fig. 5*B*). Indeed, replisome disassembly was blocked in Ubxn7-depleted extract treated with CULi, and the ubiquitylation of Mcm7 observed in Ubxn7-depleted extract at 60 min was strongly inhibited by CULi. Instead, we observed a much more gradual accumulation of ubiquitylated Mcm7, as we always do upon CULi treatment (Fig. 5*B*, compare also Fig. S6*C*). This shows that Cul2 activity is still required for replisome disassembly in the absence of Ubxn7.

Finally, we wanted to assess whether the length of chains built on Mcm7 upon Ubxn7 depletion is unusually long, suggesting uncontrolled Cul2^Lrr1^ activity or whether it is comparable to the level of ubiquitylation we observe upon blocking p97 segregase activity and replisome unloading. To this end, we inhibited p97 in Ubxn7 depleted extract (Fig. 5*C*) and could see that the level of ubiquitylation upon complete inhibition of unloading with p97i is even higher, suggesting that it is just the delay in replisome disassembly that gives Cul2^Lrr1^ more time to ligate longer ubiquitin chains.

**Figure 6:**
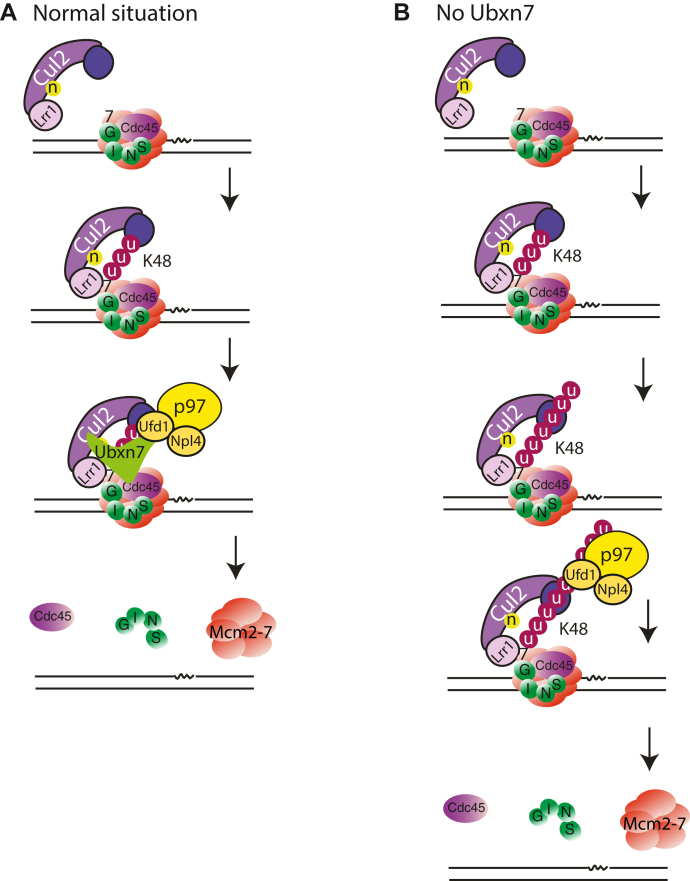
**Proposed model of Ubxn7 function.***A*, Ubxn7 is bridging Cul2^Lrr1^, ubiquitylated Mcm7, and the p97 complex, leading to efficient CMG helicase unloading. *B*, delays in replisome unloading upon lack of Ubxn7. p97 can still recognize ubiquitylated Mcm7 but the process is slower and takes longer time. CMG, Cdc45, Mcm2-7 hexamer, and GINS; DMSO, dimethyl sulfoxide.

Altogether, our data support a model whereby Ubxn7 binds to active, neddylated Cul2^Lrr1^ on chromatin to facilitate fast recruitment of the p97 complex to ubiquitylated replisomes resulting in efficient replisome unloading (Fig. 6*A*). In the absence of Ubxn7, Cul2^Lrr1^ can still bind to terminated replisome but recruitment of p97 is delayed. In the meantime, active, neddylated Cul2^Lrr1^ keeps ubiquitylating Mcm7, forming longer ubiquitin chains, which finally allow for p97 recognition and replisome disassembly (Fig. 6*B*).

## Discussion

### Ubxn7 streamlines replisome disassembly during replication termination

Our results suggest that by concomitant interactions with neddylated Cul2^Lrr1^, ubiquitylated Mcm7, and p97 complex, Ubxn7 facilitates efficient and fast unloading of terminated CMG helicases from chromatin (Fig. 6). The general mode of Ubxn7 operation in replisome disassembly during termination closely resembles the way UBXD7 regulates degradation of Hif1α in collaboration with CUL2^VHL^ and p97/UFD1/NPL4 (19, 20). It is interesting to speculate that Ubxn7 may not only bridge the three factors to facilitate recognition of terminated ubiquitylated replisomes but may also stimulate turnover of Cul2^Lrr1^ and promote its dissociation from terminated replisomes. This in turn could facilitate the unfolding of ubiquitylated Mcm7 by the p97 complex.

The ability of Ubxn7 to facilitate unloading of Mcm7 ubiquitylated with short ubiquitin chains synthesized by Cul2^Lrr1^ explains also why we observe such a strong accumulation of Mcm7 modified with short ubiquitin chains after treatment of replication reactions with CULi (Figs. 5*B* and S6*C*). CULi inhibits neddylation of cullins, so it not only slows down the activity of Cul2^Lrr1^ but also inhibits the interaction of Ubxn7 with Cul2^Lrr1^. As a result, the slowly building up chains on Mcm7 have to reach a higher threshold of length to be efficiently extracted by the p97 complex.

CULi (MLN4924) inhibits all cullin activity, and it exerts its main cytotoxic activity by inhibiting degradation of the replication licensing factor Cdt1 by CUL4^CDT2^, which leads to re-replication, checkpoint activation, and cell death (21, 23). A possibility exists, therefore, that the effects we observed upon treatment with CULi could arise from inhibition of neddylation of one of the other cullins important for DNA replication, namely Cul4 or Cul1 (24). However, in *Xenopus* egg extract, stabilization of Cdt1 is not enough to induce re-replication due to high activity of the Cdt1 inhibitor geminin (25). Moreover, the progress of nascent DNA synthesis is not affected by inhibition of ubiquitin-driven protein degradation in the *Xenopus* egg extract system (5, 26) and inhibition of Cul1 or Cul4 activity does not affect replisome disassembly process (data not shown). Finally, of all cullins, Ubxn7 binds most preferentially to CUL2 (19). Altogether, the effect exerted by CULi on Ubxn7 and the process of replisome disassembly is most likely directly through inhibition of Cul2 neddylation.

The function of Ubxn7/UBXD7 in streamlining the process of replisome disassembly may not just be through accelerated recognition of ubiquitylated substrate by the p97 segregase complex but also by increasing the rate of substrate unfolding. A recent study has shown that once the substrate is recognized, p97 starts substrate processing by unfolding one of the distal ubiquitins in the ubiquitin chain attached to the substrate. It then pulls both the unfolded ubiquitin chain and the unfolded substrate through the central channel of the hexamer (27), which leads to extraction of the substrate from complex structures. In the absence of Ubxn7, when Mcm7 is modified with long ubiquitin chains, the process of unfolding of such modified Mcm7 is likely to be slower as it is more likely for p97/Ufd1/Npl4 to bind the ubiquitin chain further away from the Mcm7 substrate, which necessitates unfolding of a longer ubiquitin chain before unfolding Mcm7 itself.

### Ubxn7 and Faf1 during replication

Immunodepletion of Ubxn7, but not Faf1, from egg extract leads to a delay in replisome disassembly (Figs. 2 and 3). The *C. elegans* homolog of Faf1, UBXN-3, has been shown to be important for CMG helicase unloading by p97 in S-phase and in mitosis (4, 11) but also to regulate other replication factors such as CDT-1 and, recently, to regulate SUMOylated factors at DNA replication forks (17, 28, 29). It is clear, therefore, that UBXN-3 plays a key role in extraction of proteins from chromatin during DNA replication in *C. elegans* embryos. There is, however, no homolog of Ubxn7 in *C. elegans*, and UBXN-3/Faf1 does not contain a UIM domain that could direct it to neddylated cullins, so it is unlikely to directly substitute for the role that Ubxn7 plays in vertebrates.

In our experiments, in the absence of Ubxn7, the unloading of the replisomes is delayed but they are still eventually unloaded (Figs. 2 and 3). At the same time, we observe accumulation of active Cul2^Lrr1^ and higher levels of ubiquitylated Mcm7 on chromatin (Fig. 3). After a delay, p97/Ufd1/Npl4 can, therefore, recognize ubiquitylated Mcm7 and extract it, possibly with help from other cofactors such as Faf1. In other organisms that have no Ubxn7, this process is also likely to be facilitated by other cofactors such as Faf1/UBXN-3. Interestingly, in an *in vitro* reconstitution system of DNA replication with purified budding yeast proteins, where no p97 cofactors were present, Mcm7 needed to be ubiquitylated with a minimum ubiquitin chain length of 5 in order to be recognized and extracted by the CDC48 complex. This process, however, worked much better with longer chains (30). The evolution of Ubxn7 to specifically link neddylated cullins with their substrates and the processing factor p97 is an additional level of regulation that ensures efficiency in p97 substrate targeting.

The role of UBXN-3 in regulating SUMOylated factors at replication forks seems to have been conserved throughout evolution as it is also the case in human immortalized cells (29). It will be very interesting to decipher the contribution of FAF1 and UBXD7 to replisome disassembly in human cells in the future.

### Importance of efficient replisome disassembly

Replisome unloading must be carefully regulated to maintain genome stability. Premature replisome unloading would likely lead to a collapse of replication forks and creation of DNA damage (31). However, defects in timely replisome disassembly are also detrimental for cells. For example, *dia2Δ* budding yeast cells, which cannot ubiquitylate Mcm7 during termination, are defective in cell cycle progression, present high levels of genomic instability, are unable to grow at low temperatures, and are sensitive to drugs that compromise replication fork progression (32, 33, 34, 35). Genetic loss of *lrr-1* in worms results in mitotically arrested *C. elegans* embryos and germ lines (36, 37). Moreover, while partial disruption of the S-phase or mitotic pathways of replisome disassembly alone had no effect on worm embryo viability, disrupting both pathways led to embryonic lethality (4). Finally, CRISPR/Cas9-mediated deletion of LRR1 or TRAIP in a number of human cell lines is lethal (38). Interestingly, a recent study suggested that efficient LRR1-mediated replisome disassembly is essential for completion of DNA replication, possibly through recycling of replisome components from early activated replication forks to late firing ones (39). With all these in mind, it is clear that timely and efficient replisome disassembly is important for the maintenance of genome stability and that Ubxn7 is pivotal for this.

## Experimental procedures

### Inhibitors

MLN4924 (A01139, Active Biochem) was dissolved in DMSO at 20 mM and added to the extract 15 min after addition of sperm nuclei at 10 μM. NMS873 (17674, Cayman Chemical Company) was dissolved in DMSO at 10 mM and added to the extract 15 min after addition of sperm nuclei at 50 μM. Caffeine (C8960, Sigma) was dissolved in water at 100 mM and added to the extract along with demembranated sperm nuclei at 5 mM. Aphidicolin was dissolved in DMSO at 8 mM and added to the extract along with demembranated sperm nuclei at 40 μM. EcoR1 (R6011, Promega) was purchased at stock 12 U/μl and added to the extract at 0.05 U/μl.

### Recombinant proteins

Recombinant His-tagged ubiquitin and ubiquitin mutants were purchased from Boston Biochem, dissolved in LFB1/50 buffer (10% sucrose, 50 mM KCl, 40 mM Hepes pH 8.0, 20 mM K phosphate pH 8.0, 2 mM MgCl_2_, 1 mM EGTA, 2 mM DTT, 1 μg/ml of each: aprotinin, leupeptin, and pepstatin) at 10 mg/ml and used at 0.5 mg/ml in *X. laevis* egg extract.

pET28a-Ubxn7, pET28a-Ubxn7-P458G, and pET28a-Ubxn7-L286E/A289Q/S293A vectors were used for protein expression in 2L of BL21-codon Plus (DE3)-RIPL (1 mM IPTG added at *A*_600_ = 0.6, followed by incubation overnight at 20 °C). Frozen bacterial pellets were lysed in LFB1/50 buffer (10% sucrose, 50 mM KCl, 40 mM Hepes pH 8.0, 20 mM K phosphate pH 8, 2 mM MgCl_2_, 1 mM EGTA, 2 mM DTT, 1 μg/ml of each: aprotinin, leupeptin, and pepstatin) and supplemented with 1 mg/ml lysozyme and BitNuclease. After sonication, the lysate was clarified by centrifugation at 14,000*g* for 30 min at 4 °C, and supernatants containing soluble proteins were then incubated with 2 ml of prewashed Super Ni-NTA affinity resin (SUPER-NINTA100, Generon) for 2 h, rotating at 4 °C. The beads were then washed 2× with 30 ml of LFB1/50 buffer and 2× with LFB1/200 (200 mM KCl), both supplemented with 20 mM imidazole. The beads were transferred to 10 ml columns (Poly-Prep Chromatography Column, Bio-Rad) and eluted with LFB1/50 supplemented with 250 mM imidazole.

To outcompete endogenous Ubxn7, recombinant Ubxn7 was used at 0.3 mg/ml in egg extract, while to rescue Ubxn7 depleted extract, it was added at 10 μg/ml.

Full-length 6xHIS-Faf1 was expressed from pET28a-Faf1 in Rosetta (DE3) pLysS competent cells (Novagen, Merck Millipore) bacteria as aforementioned. It was purified as Ubxn7 but using the following buffers: lysis buffer (500 mM NaCl, 50 mM Tris–HCl pH 8.0, 2 mM MgCl_2_, 10% glycerol, 0.1% Triton, 1 μg/ml of each: aprotinin, leupeptin, and pepstatin, 1 mg/ml lysozyme). After incubation, beads were washed with lysis buffer supplemented with 20 mM imidazole. Beads were eluted with lysis buffer supplemented with 250 mM imidazole.

*X. laevis* GINS was expressed in BL21 (DE3) competent *Escherichia coli* cells (C2527H, New England Biolabs) bacteria. The isolated cell pellets were then resuspended into 50 mM Tris pH 7.4, 500 mM NaCl, 0.1 mM PMSF (Sigma), and protease inhibitor tablets (11836170001, Roche). Samples were sonicated for 3X 1 min, 2 s pulses - 50% duty cycle and supplemented with 1 mg/ml lysozyme. The suspension was then incubated on rocker at 4 °C for 1 h and then clarified by centrifugation at 20,000*g* for 30 min at 4 °C, followed by filtration through 0.22 μm polyethersulfone filters (Millipore). The supernatant was then passed through 5 ml His-Trap HP column (Cytiva) connected to AKTAprime plus (GE). Gradient elutions were performed with imidazole containing buffer (50 mM Tris pH 7.4, 500 mM NaCl, 500 mM imidazole, and 0.1 mM PMSF). *X. laevis* GINS fractions were eluted with ∼79 to 134 mM imidazole. All fractions were pooled and stored in −80 °C.

### Antibodies

α-PCNA (P8825) and α-HIS (H1029) were purchased from Sigma; α-Cul2 (EPR3104) was purchased from Abcam; α-p97 (65278) was purchased from Progen Biotechnik, anti-P-Chk1 (S345) from Cell Signaling, γ-H2AX (4418-APC-020, Trevigen).

Affinity purified α-Cdc45, α-Psf2 (40), α-Mcm3 (41), α-LRR1 (S962D) and α-Cul2 (SA206) (4), and α-Mcm7 (6) were previously described. *Xenopus* Ufd1 antibody was a kind gift from Prof Stemmann’s lab (13).

*Xenopus* full-length p97, Ubxn7, Faf1, and GINS proteins were purified as described previously (6, 13) and antibodies raised against such prepared antigens in sheep (p97, GINS, Ubxn7) or rabbit (Faf1). The resulting antibody sera were purified in-house against the purified antigen. The specificity of each new antibody is presented in Fig S7.

### *X. laevis* egg extract preparation

All of the work with *X. laevis* was approved by Animal Welfare and Ethical Review Body (AWERB) at University of Birmingham and approved by UK Home Office in form of Project License issued for Dr Agnieszka Gambus. *X. laevis* egg extract was prepared as previously described (42).

### DNA synthesis assay

Interphase *X. laevis* egg extract was supplemented with 10 ng/μl of demembranated sperm nuclei and incubated at 23 °C for indicated time. Synthesis of nascent DNA was then measured by quantification of α^32^P-dATP incorporation into newly synthesized DNA, as described before (42). The extract contains endogenous deoxynucleoside triphosphate pools of ∼50 μM (43). The total amount of DNA synthesized, expressed as nanogram DNA/μl extract, can then be calculated by multiplying percent total ^32^P incorporated by a factor of 0.654 (43). This calculation assumes an average molecular weight of 327 Da for deoxynucleoside monophosphate and equal quantities of all four deoxynucleoside triphosphates incorporated into DNA (weight of deoxynucleoside monophosphate incorporated in ng/μl = percent total ^32^P incorporated/100 × 50 × 10^−6^ × 4 × 327 × 10^3^) (43).

### Chromatin isolation time course

Interphase *X. laevis* egg extract was supplemented with 10 to 15 ng/μl of demembranated sperm DNA and subjected to indicated treatments. The reaction was incubated at 23 °C for indicated length of time when chromatin was isolated in ANIB100 buffer (50 mM Hepes pH 7.6, 100 mM KOAc, 10 mM MgOAc, 2.5 mM Mg-ATP, 0.5 mM spermidine, 0.3 mM spermine, 1 μg/ml of each aprotinin, leupeptin, and pepstatin, 25 mM β-glycerophosphate, 0.1 mM Na_3_VO_4,_ 0.2 μM microcystin-LR, and 10 mM 2-chloroacetamide [Merck]) as described previously (42).

During the chromatin isolation procedure, a sample without addition of sperm DNA (no DNA) is processed in an analogous way, usually at the end of the time course, to serve as a chromatin specificity control. The bottom of the PAGE gel on which the chromatin samples were resolved is cut off and stained with colloidal Coomassie (SimplyBlue, Life Technologies) to stain histones which provide loading controls and indications of sample contamination with egg extract (cytoplasm).

### Nuclei isolation for Chk1 phosphorylation

The nuclei isolation was performed as previously described (5).

### Immunoprecipitation from egg extract

Twenty microliter of egg extract per IP was induced into interphase, and the extract was then supplemented with 4 vol of LFB1/50 buffer (10% sucrose, 50 mM KCl, 40 mM Hepes pH 8, 20 mM K phosphate pH 8, 2 mM MgCl_2_, 1 mM EGTA, 2 mM DTT, 1 μg/ml of each: aprotinin, leupeptin, and pepstatin). The diluted extract was cleared of insoluble material by 15 min centrifugation in a microfuge at 4 °C, 16k rcf. About 100 μl of diluted extract was supplemented with 1 μg of affinity purified Ubxn7, p97, or IgG from sheep serum (I5131, Sigma) and incubated on ice for 1 h with sporadic mixing. Twenty microliter of prewashed Protein G Dynabeads (10004D, Life Technologies) were added to each IP sample and incubated for 1 h at 4 °C with rotation. After incubation the beads were washed 3× with LFB1/50 and boiled in NuPAGE LDS loading buffer (Life Technologies).

### Immunoprecipitation from chromatin

Hundred microliter of egg extract per IP was induced into interphase and mixed with 10 to 15 ng/μl demembranated sperm nuclei and optionally supplemented with the indicated treatments. The reaction was incubated at 23 °C for the indicated time. Chromatin was isolated in ANIB100 (50 mM Hepes pH 7.6, 100 mM KOAc, 10 mM MgOAc, 2.5 mM Mg-ATP, 0.5 mM spermidine, 0.3 mM spermine, 1 μg/ml of each aprotinin, leupeptin, and pepstatin, 25 mM β-glycerophosphate, 0.1 mM Na_3_VO_4,_ 0.2 μM microcystin-LR, and 10 mM 2-chloroacetamide), and the chromatin pellets were resuspended in the same volume of original extract of ANIB100 containing 20% sucrose. Protein complexes were released from chromatin by digestion with 2 U/μl of Benzonase nuclease (E1014-25KU, Sigma) and sonicated for 5 min using a Diagenode sonicator with following settings: 15s on, 15s off, medium setting. The insoluble fraction was then spun in a microfuge at 4 °C, 10 min, 16k rcf.

Prepared beads,

(1) 30 μl of Dynabeads M-270 epoxy (14302D, Life Technologies) coupled covalently to 20 μg of affinity purified p97 antibody, affinity purified Ubxn7 antibodies, or IgG from sheep serum (I5131, Sigma) and
(2) 30 μl of Dynabeads Protein G (10004D, Life Technologies) covalently coupled to 6 μg of affinity purified p97 antibody, affinity purified Ubxn7, affinity purified Cul2, or IgG from sheep serum (I5131, Sigma) using BS3 crosslinker (S5799, Sigma),

were incubated with 100 μl digested chromatin at 4 °C for 1 to 2 h with rotation. Following the incubation time, beads were washed for 5 min rotating at 4 °C twice with ANIB100, once with ANIB100 containing an additional 0.1% Triton X-100, and finally twice with ANIB100 buffer. Each sample was prepared by boiling in 30 μl of 2× NuPAGE LDS loading buffer (Life Technologies) for 5 min.

### Immunoprecipitation of p97 and MS

About 3.75 ml of *X. laevis* egg extract was activated and supplemented with 10 ng/μl of demembranated sperm DNA, 50 μM p97 inhibitor NMS873, and incubated at 23 °C for 60 min. Chromatin was isolated in ANIB/100 buffer. Immunoprecipitation of p97 was performed as described previously (4), and the immunoprecipitated material was analyzed by MS with Dr Richard Jones from MS Bioworks LLC.

#### Sample preparation

Each sample was run on a 5% to 20% gradient gel (Invitrogen) for 1 cm and cut into 10 bands. Samples were submitted preplated for 10 fraction analysis. Gel pieces were processed using a robot (Progest, DigiLab) with the following protocol:

(1) Washed with 25 mM ammonium bicarbonate followed by acetonitrile.

(2) Reduced with 10 mM DTT at 60 °C followed by alkylation with 50 mM iodoacetamide at room temperature.

(3) Digested with trypsin (Promega) at 37 °C for 4 h.

(4) Quenched with formic acid and the supernatant was analyzed directly without further processing.

#### MS

The gel digests were analyzed by nano LC/MS/MS with a Waters M-class HPLC system interfaced to a ThermoFisher Fusion Lumos. Peptides were loaded on a trapping column and eluted over a 75 μm analytical column at 350 nl/min; both columns were packed with Luna C18 resin (Phenomenex). A 30 min gradient was employed. The mass spectrometer was operated in data-dependent mode, with MS and MS/MS performed in the Orbitrap at 60,000 FWHM resolution and 15,000 FWHM resolution, respectively. Advanced peak determination was turned on. The instrument was run with a 3 s cycle for MS and MS/MS. Proteome Discoverer v1.4 (ThermoFisher; www.thermofisher.com) was used for peak generation.

#### Data processing

Data were searched using a local copy of Mascot (Matrix Science; version 2.8.0.1) with the following parameters:

Enzyme: Trypsin Fully Specific

Database: Uniprot *Xenopus* (forward and reverse appended with common contaminants) released on 04/15/2014. 79,274 (including reverse and CON) entries in the database were searched.

Fixed modification: Carbamidomethyl (C)

Variable modifications: Oxidation (M), Acetyl (Protein N-term), Deamidation (NQ), GlyGly (K), Phospho (STY)

Mass values: Monoisotopic

Peptide Mass Tolerance: 10 ppm

Fragment Mass Tolerance: 0.02 Da

Max Missed Cleavages: 2

Mascot DAT files were parsed into the Scaffold (version Scaffold_5.1.0, Proteome Software Inc) software for validation, filtering, and to create a nonredundant list per sample. Data were filtered with 1% protein and peptide false discovery rate (FDR) and requiring at least two unique peptides per protein.

Peptide identifications were accepted if they could be established at greater than 34.0% probability to achieve an FDR less than 1.0% by the Percolator posterior error probability calculation (44). Protein identifications were accepted if they could be established at greater than 99.0% probability to achieve an FDR less than 1.0% and contained at least two identified peptides. Protein probabilities were assigned by the Protein Prophet algorithm (45). Proteins that contained similar peptides and could not be differentiated based on MS/MS analysis alone were grouped to satisfy the principles of parsimony.

### Immunodepletion

Ubxn7 immunodepletions were performed using Dynabeads protein G (10004D, Life Technologies) coupled to antibodies against Ubxn7 or nonspecific sheep IgGs (I5131, Sigma), with two rounds of 1 h incubation at 4 °C. The Ubxn7 antibodies were coupled at 600 μg per 1 ml of beads. Effective immunodepletion required two rounds of 1 h incubation of egg extract with antibody coupled beads at 50% beads ratio.

Faf1 immunodepletions were performed using Dynabeads protein A (10002D, Life Technologies) coupled to *Xenopus* Faf1 antibodies raised in rabbit and affinity purified or nonspecific rabbit IgG (I5006, Sigma). The Faf1 antibodies were coupled at 600 μg per 1 ml of beads. Effective immunodepletion required three rounds of 40 min incubation of egg extract with antibody coupled beads at 50% beads ratio.

### HIS-pull down from egg extract

Thirty microliter of interphase egg extract per pull down was supplemented with 10 ng/μl of demembranated sperm nuclei and optionally supplemented with LFB1/50 buffer or 0.3 mg/ml of recombinant Ubxn7^ΔUBX^, Ubxn7^ΔUIM^, or Ubxn7^wt^ proteins. The replication reaction was stopped with LFB1/50 buffer supplemented with 0.1% Triton X-100 and chloroacetamide in the middle of the S-phase. The samples were sonicated for 5 min using the Diagenode cold water sonicator with following settings: 15s on, 15s off, medium settings. Insoluble material was clarified for 10 min at 4 °C at 16k rcf and incubated with 60 μl Dynabeads HIS-tag isolation (10104D, Invitrogen) for 2 h with rotation at 4 °C. Beads were subsequently washed two times with LFB1/50 buffer supplemented with 0.1% Triton X-100 and chloroacetamide. Pulled-down HIS-tagged proteins were eluted by boiling the beads in 30 μl of 2× NuPAGE LDS loading buffer for 5 min.

### Western blot quantification

The quantification of Western blots is provided to indicate reproducibility of trends in experiments rather than to provide absolute values of increases or decreases in a signal. The quantified experiments were performed in different preparations of extracts and independently immunodepleted extracts to confirm that observed phenotypes are not specific for one extract preparation. As a result, the extracts differ slightly in their kinetics of DNA replication reaction, which can affect the levels of detected proteins on chromatin at the same time points between different experiments. They do, however, all reproducibly show the same trend of change across the experiments.

The density of pixels of each band of the Western blot and scanned stained histones within the gel were quantified using ImageJ software (National Institute of Health; https://imagej.net/software/imagej/). The numeric value in arbitrary units for each band was normalized to loading control (bands of Coomassie stained histones). The analysis of control and treatment samples was always done together and the fold difference between them calculated. Fold change from a number of repeated experiments is plotted on the graphs with mean value and SEM as calculated by GraphPad PRISM.

## Data availability

The mass spectrometry proteomics data have been deposited to the ProteomeXchange Consortium *via* the PRIDE repository with the dataset identifier:

**Project Name:** Immunoprecipitation of p97 from *X. laevis* S-phase chromatin

**Project accession:** PXD029705

**Project DOI:** 10.6019/PXD029705

## Supporting information

This article contains supporting information.

## Conflict of interest

The authors declare that they have no conflicts of interest with the contents of this article.

## Supporting information

Supplementary fig 7

Supporting Information Table p97 IP identified proteins
