# Supplementary material for: The p97 segregase cofactor Ubxn7 facilitates replisome disassembly during S-phase
Source: J Biol Chem. 2022 Jul 4;298(8):102234. doi: 10.1016/j.jbc.2022.102234 (PMC9358472; doi:10.1016/j.jbc.2022.102234)
Supplement: Supplementary fig 1 [file mmc1.pdf]

Tarcan et all Supp Figure 1

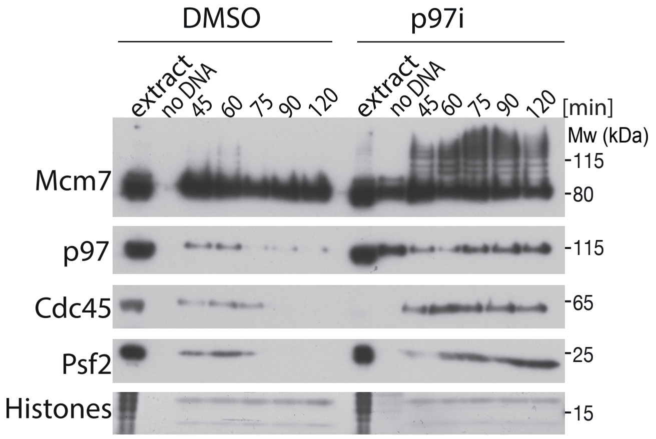

Supplementary Figure 1

p97 accumulates on chromatin upon inhibition of its ATPase activity. Interphase egg extract was supplemented with DMSO or p97i and chromatin samples isolated at indicated timepoints during replication reaction. Chromatin samples were analysed by western blotting with indicated antibodies as in Figure 1A. Presence of a ladder of bands in -DNA control in p97i indicates contamination with cytoplasm in this particular sample. See also Figure 4A for alternative western blot. CMG components (Cdc45 and Psf2) unloading is inhibited with p97i and ubiquitylated forms of Mcm7 accumulate on chromatin when p97 is not active.
