# Supplementary material for: The p97 segregase cofactor Ubxn7 facilitates replisome disassembly during S-phase
Source: J Biol Chem. 2022 Jul 4;298(8):102234. doi: 10.1016/j.jbc.2022.102234 (PMC9358472; doi:10.1016/j.jbc.2022.102234)
Supplement: Supplementary fig 2 [file mmc2.pdf]

Tarcan et all Supp Figure 2

A

B

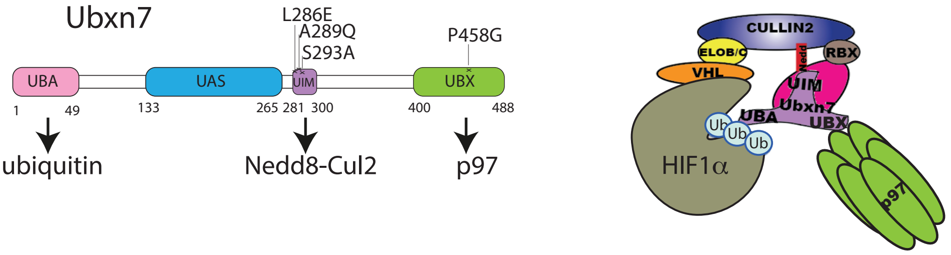

C

```
X.l.   MEKDGG-----VSQQLLREFTAITGATDSVAKHMLEACNHNLEMAVTMFLDGGGIPDEPS   55
H.s.   MAAHGGSAASSALKGLIQQFTTITGASESVGKHMLEACNNNLEMAVTMFLDGGGIAEEPS   60
       *  .**     . : *:::**:****::**.********:*************** :***

X.l.   TSSAGSSTARPAPDLSDDVRAPIPQKQEILVEPE-IFGAPKRRRPARSIFDGFRDFQTET   114
H.s.   TSSASVSTVRP--HTEEEVRAPIPQKQEILVEPEPLFGAPKRRRPARSIFDGFRDFQTET   118
       ****. **.**  . .::**************** :************************

X.l.   IRQEQELRNGGAVDKKLTTLADLFRPPIDLMHKGSFETAKQFGQLHNKWLMINIQNVQDF   174
H.s.   IRQEQELRNGGAIDKKLTTLADLFRPPIDLMHKGSFETAKECGQMQNKWLMINIQNVQDF   178
       ************:***************************: **::**************

X.l.   ACQCLNRDIWSNDAIKTLIREHFIFWQVYHDSEEGQRYIQFYKLPEFPYVSILDPRTGQK   234
H.s.   ACQCLNRDVWSNEAVKNIIREHFIFWQVYHDSEEGQRYIQFYKLGDFPYVSILDPRTGQK   238
       ********:***:*:*.:************************** :**************

X.l.   LVEWHKLDPNSFVEQVTGFLGEHGQLDGLSSSPPKKRLRSESLIDASEDSQLEAAIRASL   294
H.s.   LVEWHQLDVSSFLDQVTGFLGEHGQLDGLSSSPPKKCARSESLIDASEDSQLEAAIRASL   298
       *****:** .**::**********************  **********************

X.l.   QETHFDSAVNKETPRREEESESELYSGSEEFISVCGSDHEEDEERPVIESSAEPDSCGKS   354
H.s.   QETHFDSTQTKQDSRSDEESESELFSGSEEFISVCGSDEEEEVENLA-----------KS   347
       *******: .*:  * :*******:*************.**: *. .           **

X.l.   KKSTSKDPNHRKEEVGRTH--PVVVEE---TSNHQSPE---DTASSEEHHRESGCSVAVS   406
H.s.   RKSPHKDLGHRKEENRRPLTEPPVRTDPGTATNHQGLPAVDSEILEMPPEKADGVVEGID   407
       :**  ** .*****  *    * *  :   ::***.     .   .   .: .*   .:.

X.l.   DNGPKAKLMLRYPDGKKEQIALPEQAKLLALVRHVQLKGYPNERFELLTNFPRRKLSHLD   466
H.s.   VNGPKAQLMLRYPDGKREQITLPEQAKLLALVKHVQSKGYPNERFELLTNFPRRKLSHLD   467
        *****:*********:***:***********:*** ***********************

X.l.   YDVTLQEAGLCPQETVFVQERN   488
H.s.   YDITLQEAGLCPQETVFVQERN   489
       **:*******************
```

Supplementary Figure 2

(A) Model of *Xenopus* Ubxn7 structure. The key domains are highlighted together with the mutations incorporated to disrupt UIM and UBX interactions. **(B)** Model of Ubxn7 function during Hif1α processing. **(C)** Comparison of *Xenopus laevis* and human Ubxn7 protein sequence. The domains are highlighted in the same colour as in (A). Amino acids mutated in Ubxn7$^{\Delta UBX}$ and Ubxn7$^{\Delta UIM}$ mutants are highlighted in red.
