# Supplementary material for: The p97 segregase cofactor Ubxn7 facilitates replisome disassembly during S-phase
Source: J Biol Chem. 2022 Jul 4;298(8):102234. doi: 10.1016/j.jbc.2022.102234 (PMC9358472; doi:10.1016/j.jbc.2022.102234)
Supplement: Supplementary fig 3 [file mmc3.pdf]

Tarcan et al Supp Figure 3

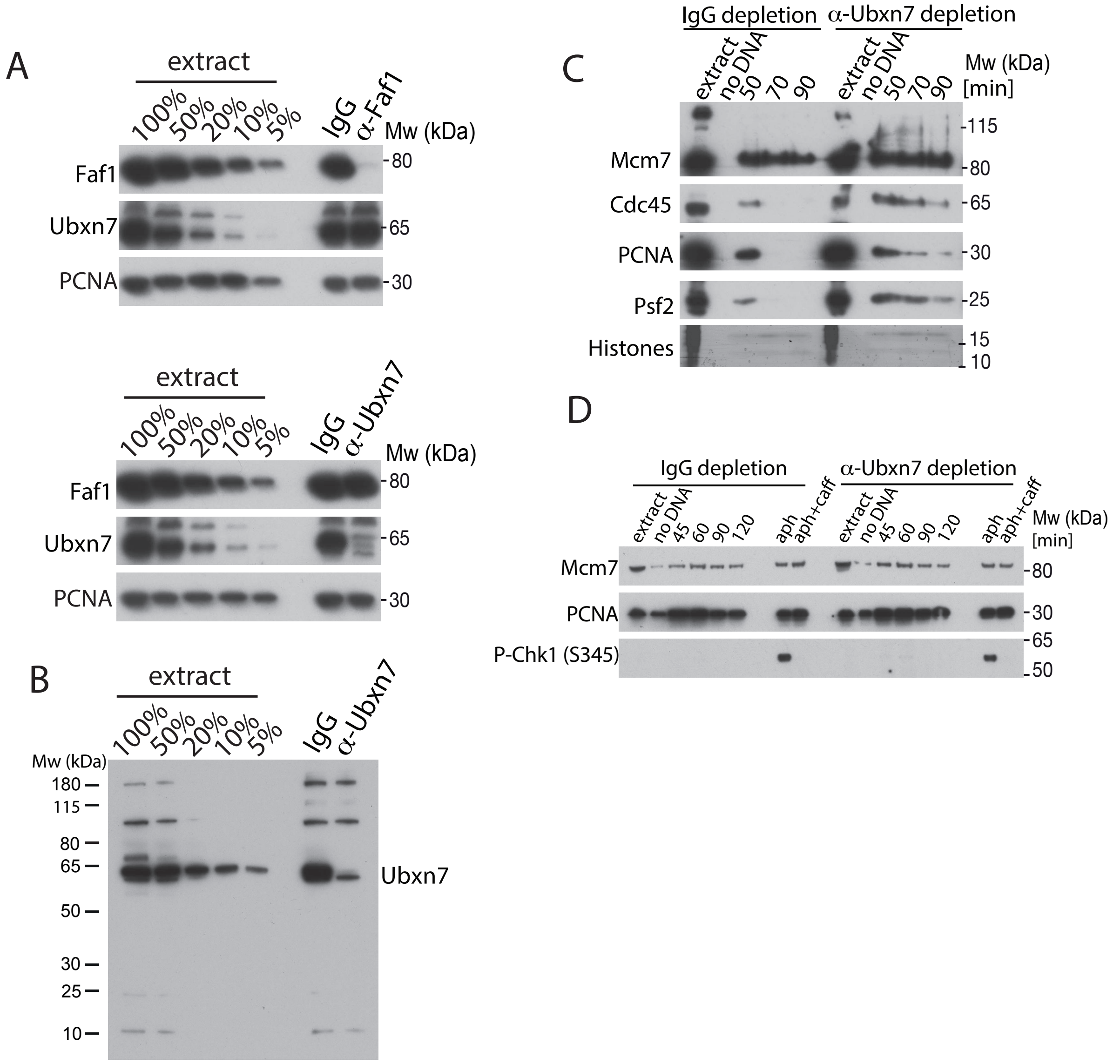

Supplementary Figure 3

(A) Immunodepletion of Ubxn7 or Faf1 does not co-deplete each other. Faf1 and Ubxn7 were immunodepleted independently as described in materials and methods. The level of remaining proteins in the egg extract was analysed through western blotting of a series of dilutions of egg extract and samples of IgG-, Ubxn7- and Faf1-depleted extracts. PCNA serves as an example of protein that is not affected by either immunodepletion. **(B)** Immunodepletion of Ubxn7 does not immunodeplete any other bands. Whole gel of Ubxn7 immunodepletion. **(C)** Immunodepletion of Ubxn7 delays replisome disassembly. An alternative example of experiment in Figure 2C. **(D)** Immunodepletion of Ubxn7 does not lead to checkpoint activation and Chk1 phosphorylation. Nuclei were isolated at indicated timepoints during replication reaction in IgG- or Ubxn7-depleted extracts. Nuclei samples were analysed by western blotting with indicated antibodies. As a positive control a sample of each extract was treated with polymerase inhibitor aphidicolin (inhibitor of Family B of polymerases which stops nascent DNA synthesis, leads to uncoupling of helicase and polymerase and induces checkpoint activation) or aphidicolin and caffeine (inhibitor of both ATR and ATM kinases which start S-phase checkpoint response).
