# Supplementary material for: The p97 segregase cofactor Ubxn7 facilitates replisome disassembly during S-phase
Source: J Biol Chem. 2022 Jul 4;298(8):102234. doi: 10.1016/j.jbc.2022.102234 (PMC9358472; doi:10.1016/j.jbc.2022.102234)
Supplement: Supplementary fig 4 [file mmc4.pdf]

Tarcan et al Supp Figure 4

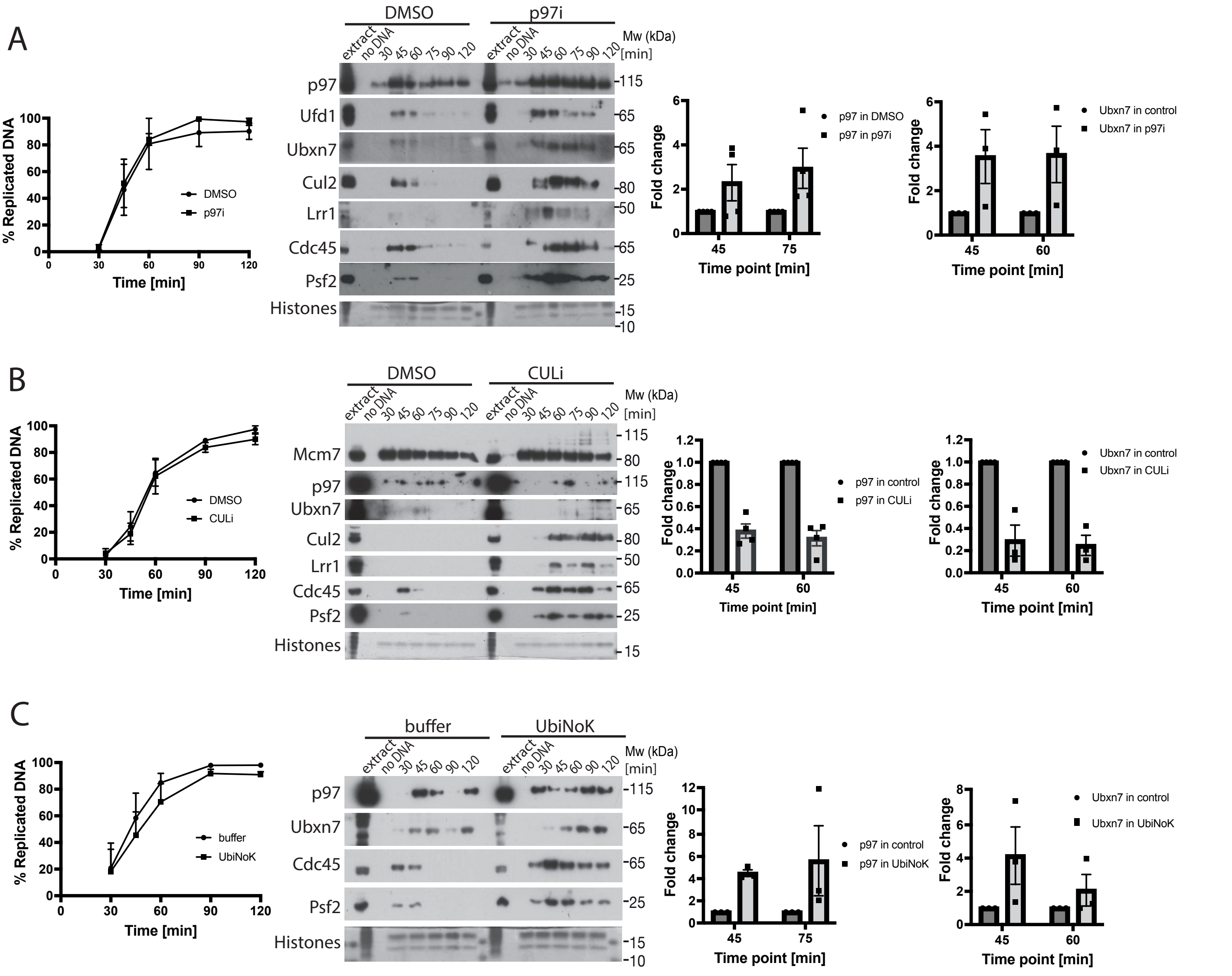

Supplementary Figure 4

(A) Addition of p97i to egg extract does not affect extract's ability to synthesise DNA. Interphase egg extract was supplemented with DMSO or p97i and the incorporation of α^{32}P-dATP into newly synthesised DNA was measured at indicated times. Mean of n=2 for p97i with SEM. p97 and Ubxn7 accumulate on chromatin upon p97i treatment. An alternative experiment presented to the one in Figure 4A. The level of p97 and Ubxn7 bound to chromatin at 45, 60 or 75 min was quantified in DMSO and p97i treated extract. 45 min timepoint represents time when replisomes are present on chromatin in control and treatment sample, while at 60/75 min replisomes are mostly unloaded in control sample. Fold increase in p97i over control is presented as a mean value with individual value points (n=4 for p97 and n=3 for Ubxn7). **(B)** Addition of CULi to egg extract does not affect extract's ability to synthesise DNA. Interphase egg extract was supplemented with DMSO or CULi and the incorporation of α^{32}P-dATP into newly synthesised DNA was measured at indicated times. Mean of n=4 with SEM. p97 and Ubxn7 decreased on chromatin upon CULi treatment - quantification of an experiment in Figure 4B as above (n=4). **(C)** Addition of 6His-UbiNOK to egg extract does not affect extract's ability to synthesise DNA. Analysed as above. Quantification of p97 and Ubxn7 accumulation on chromatin upon 6HIS-UbiNOK treatment as in Figure 4C (n=3).
