# Supplementary material for: The p97 segregase cofactor Ubxn7 facilitates replisome disassembly during S-phase
Source: J Biol Chem. 2022 Jul 4;298(8):102234. doi: 10.1016/j.jbc.2022.102234 (PMC9358472; doi:10.1016/j.jbc.2022.102234)
Supplement: Supplementary fig 5 [file mmc5.pdf]

Tarcan et all Supp Figure 5

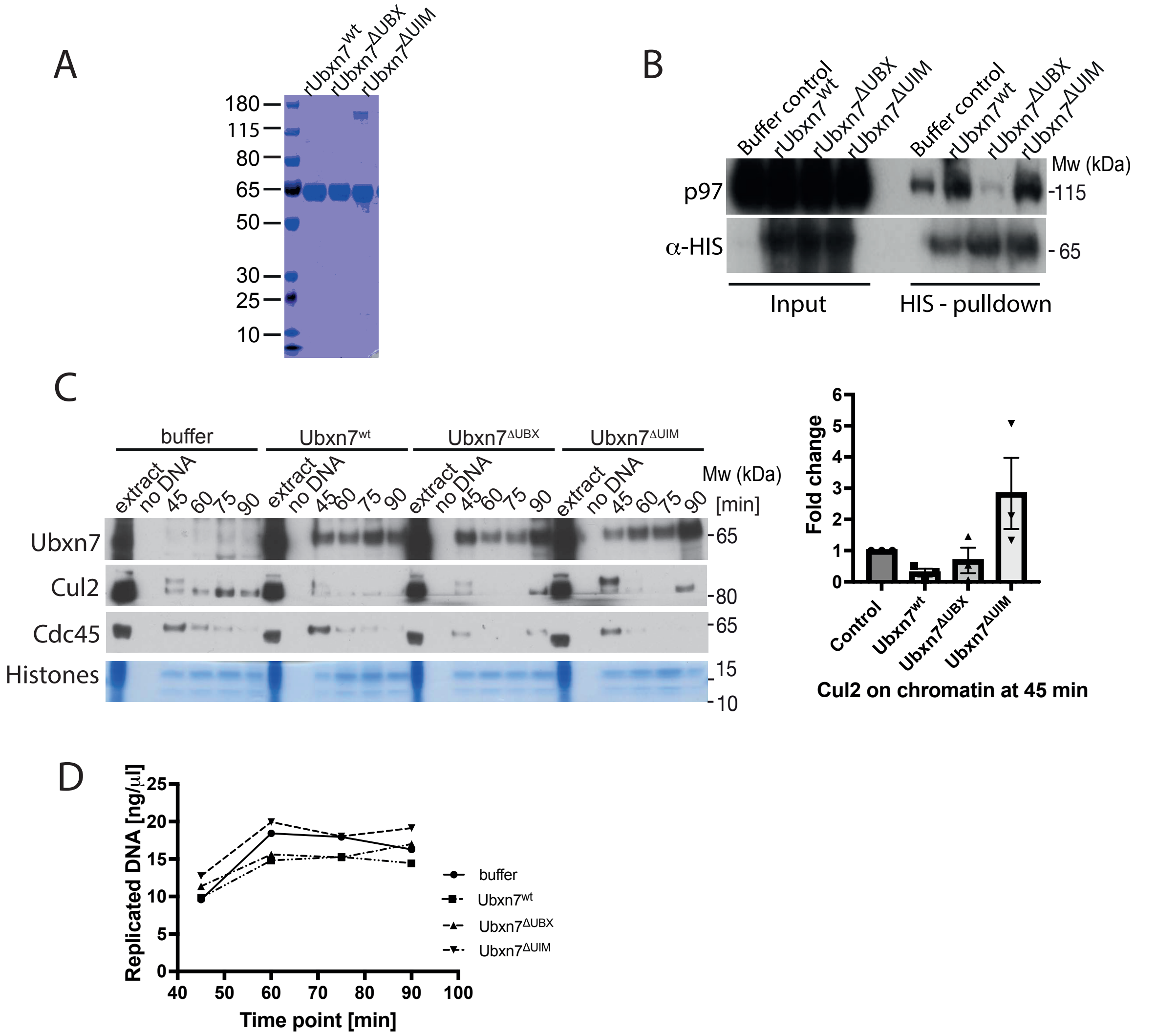

Supplementary Figure 5.

(A) Recombinant 6xHIS-Ubxn7, 6xHIS-Ubxn7$^{\Delta UBX}$ or 6xHIS-Ubxn7$^{\Delta UIM}$ were purified and equal quantity run on a PAGE gel and stained with coomassie. **(B)** Ubxn7$^{\Delta UBX}$ cannot interact with p97. Interphase egg extract was supplemented with recombinant 6xHIS-Ubxn7, 6xHIS-Ubxn7$^{\Delta UBX}$ or 6xHIS-Ubxn7$^{\Delta UIM}$ and the recombinant proteins were pulled out from replicating egg extract in the middle of S-phase. The ability of recombinant proteins to interact with p97 was analysed by western blotting.
(C) Interphase egg extract was supplemented with Ubxn7 or mutants as in (A) and chromatin samples isolated at indicated timepoints during replication reaction. Chromatin samples were analysed by western blotting with indicated antibodies (left). The level of Cul2 bound to chromatin at 45 min of replication reaction was quantified (n=3). Fold change over buffer control is presented. Individual points, mean and SEM. **(C)** Addition of high concentration of rUbxn7 or its mutants to the egg extract does not inhibit egg extract ability to replicate DNA. LFB1/50 buffer or 6HIS-Ubxn7, 6HIS-Ubxn7$^{\Delta UBX}$ or 6HIS-Ubxn7$^{\Delta UIM}$ at 0.3 mg/ml final concenetration. Extract ability to incorporate α-^{32}PdATP into nascent DNA was quantified.
