# Supplementary material for: The p97 segregase cofactor Ubxn7 facilitates replisome disassembly during S-phase
Source: J Biol Chem. 2022 Jul 4;298(8):102234. doi: 10.1016/j.jbc.2022.102234 (PMC9358472; doi:10.1016/j.jbc.2022.102234)
Supplement: Supplementary fig 6 [file mmc6.pdf]

Tarcan et all Supp Figure 6

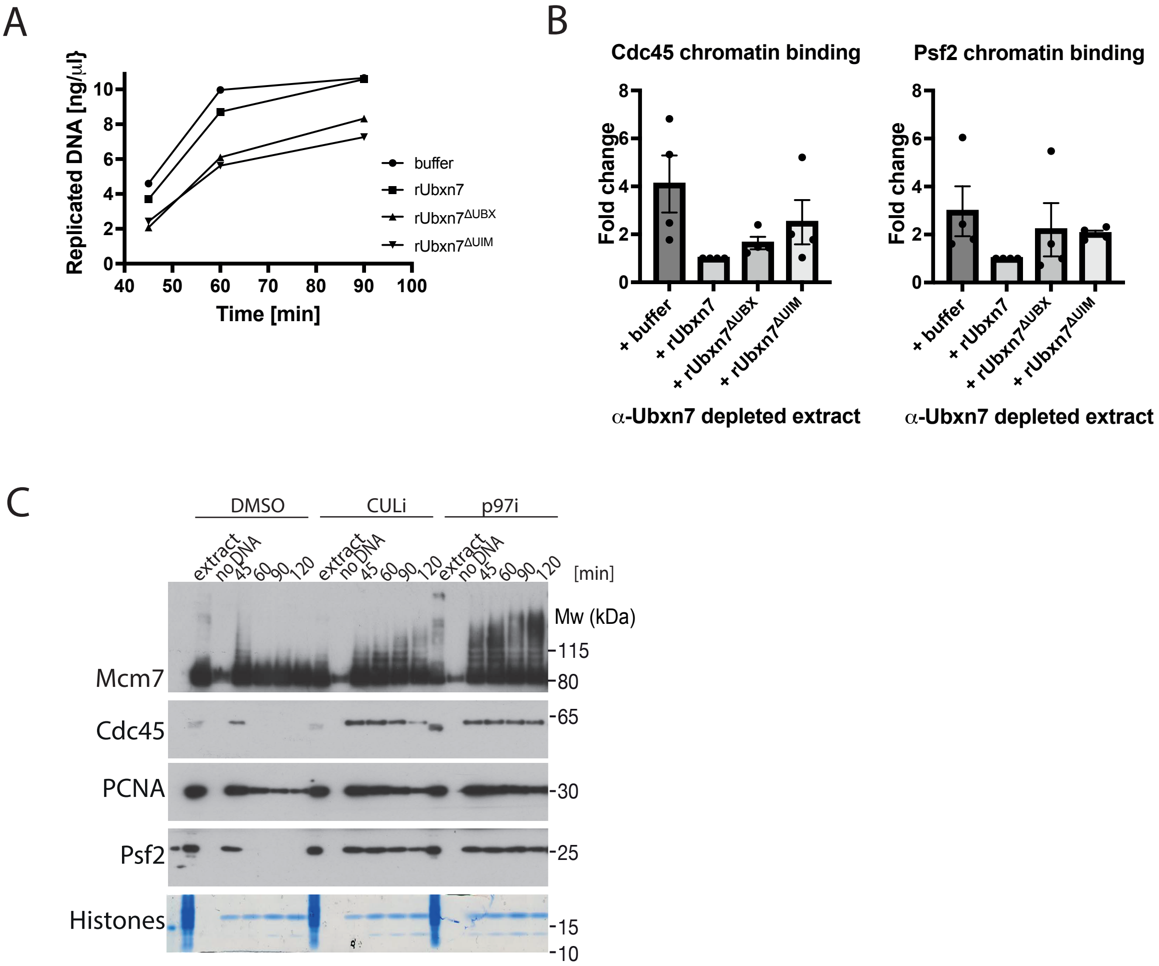

Supplementary Figure 6.
(A) Addition of recombinant 6xHIS-Ubxn7, 6xHIS-Ubxn7$^{\Delta UBX}$ or 6xHIS-Ubxn7$^{\Delta UIM}$ to rescue Ubxn7-depleted extract does not inhibit extracts ability to synthesise DNA. Ubxn7 depleted extract was supplemented with 6xHIS-Ubxn7, 6xHIS-Ubxn7$^{\Delta UBX}$ or 6xHIS-Ubxn7$^{\Delta UIM}$ and extract ability to incorporate α-^{32}PdATP into nascent DNA quantified. **(B)** UBX and UIM domains are needed for Ubxn7 activity. Quantification of experiment in Figure 5A. The chromatin bound Cdc45 and Psf2 at 75 min of replication reaction in Ubxn7-depleted extract supplemented with recombinant 6xHIS-Ubxn7, 6xHIS-Ubxn7$^{\Delta UBX}$ or 6xHIS-Ubxn7$^{\Delta UIM}$ were quantified, n=4. Individual points, mean and SEM are presented. **(C)** Mcm7 accumulates on chromatin modified with short ubiquitin chains upon cullin inhibition. Interphase egg extract was supplemented with DMSO, CULi (MLN4924) or p97i (NMS873) and chromatin was isolated during replication reaction at indicated time points after sperm DNA addition. Chromatin samples analysed as in Figure 1A.
