# Supplementary material for: The p97 segregase cofactor Ubxn7 facilitates replisome disassembly during S-phase
Source: J Biol Chem. 2022 Jul 4;298(8):102234. doi: 10.1016/j.jbc.2022.102234 (PMC9358472; doi:10.1016/j.jbc.2022.102234)
Supplement: Supplementary fig 7 [file mmc7.pdf]

Tarcan et all Supp Figure 7

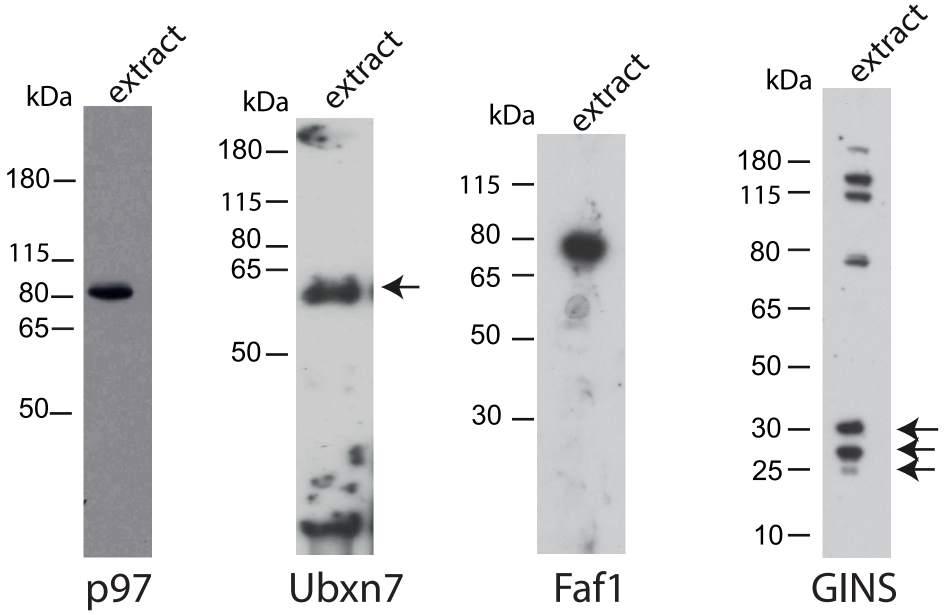

Supplementary Figure 7
All of the antibodies raised for this study. For all of the antibodies, a 0.5 μl
sample of egg extract was resolved on PAGE and immunblotted using the new affinity purified antibody.
